# Communication-free fault-tolerant control of distributed DC microgrid against sensor faults

**DOI:** 10.1038/s41598-026-41518-y

**Published:** 2026-03-10

**Authors:** Mohammed Said Ouahabi, Abdelhafid Benyounes, Said Barkat, Syphax Ihammouchen, Toufik Rekioua, Mustapha Habib, Abdullah K. Alanazi, Abdelaziz Rabehi

**Affiliations:** 1 Electrical Engineering Laboratory, Faculty of Technology, University of M’sila, M’sila, 28000 Algeria; 2 Laboratoire de Technologie industrielle et de l’Information, University of Bejaia, Bejaia, 06000 Algeria; 3 Division of Building Technology and Design, Department of Civil and Architectural Engineering, KTH Royal Institute of Technology, Stockholm, 11428, Sweden; 4 Department of Chemistry, College of Science, Taif University, Taif, Saudi Arabia; 5 Telecommunications and Smart Systems Laboratory, Faculty of Sciences and Technology, University of Djelfa, Djelfa, 17000 Algeria

**Keywords:** DC Microgrids, Fault-tolerant control, Sensor fault, Sensor failure, Unknown input observer- Passivity control, Energy science and technology, Engineering, Mathematics and computing

## Abstract

DC Microgrids are becoming increasingly popular for their efficiency and suitability for integrating renewable energy source and energy storage systems. However, unexpected sensor faults can severely compromise voltage regulation, current sharing, and overall system stability, posing a risk, especially for critical applications. Existing resilient control schemes for DC Microgrids often relies on hardware redundancy, multiple observers, or communication-based fault mitigation, leading to slow fault mitigation, increased cost, complexity, and vulnerability to cyber threat. To address the limitations of existing methods this paper proposes real-time reconfiguration framework to tolerate adverse sensor faults in islanded DC Microgrids. The proposed scheme leverages a single Proportional Integral Unknown Input Observer (PI-UIO) to reconstruct sensor faults and reconfigure a decentralized Passivity Based Control (PBC) at the primary level and a distributed consensus based current sharing controller at the secondary level. Unlike conventional methods, the proposed scheme operates autonomously without communication, thus enhancing the scalability, reliability and resilience against cyberattacks. Moreover, the design of the PI-UIO and PBC is achieved with decentralized parameters to enable seamless plug-and-play integration. Extensive simulation and real time simulation results validate the effectiveness and superiority of the proposed FTC framework compared with the recent methods.

## Introduction

Recently, DC Microgrids have emerged as a promising approach for integrating distributed and renewable energy sources, along with distributed energy storage systems. Their growing attention stems from several advantages over AC Microgrids, including higher efficiency, simpler control, and the elimination of reactive components^1,2^. DC Microgrids are currently being deployed in various applications such as ships, data centers, and energy storage backup systems^3–6^. Typically, the DC Microgrid consists of multiple distributed generation units (DGUs) interconnected by power distribution lines. Each DGU includes a DC voltage source, often generated by a renewable energy resource such as photovoltaic, a DC-DC converter, and a resistive-inductive-capacitive filter, and feeds some loads at its point of common coupling (PCC)^7^. In this, the primary control challenges include maintaining system stability, regulating each DGU’s output voltage to its designated set point to ensure the safe and reliable operation of connected loads, and achieving proportional load sharing^8,9^.

To address these challenges, several control schemes have been proposed in the literature. Centralized control requires a central controller to gather the system-wide information and send control commands to all DGUs via high bandwidth communication links. However, this approach suffers from its high susceptibility to single-point failures and comes with high implementation costs. Given the highly distributed nature of Microgrids, centralized control may not be a valuable solution. Especially in terms of scalability, as the computational and communication burden of these architectures increases with the larger size of Microgrids^10,11^.

On the other hand, traditional *V-I droop*-based control emerges as the widely adopted decentralized approach due to the simplicity and independence of any communications. Despite its convenience, simplicity, and inexpensiveness, droop-based control suffers from voltage deviation, biased power-sharing instability issues, and poor transient responses to large and fast load variations^12–14^.

Alternatively, distributed control leverages the sparse communication among neighboring DGUs, showing excellent autonomy, scalability, higher reliability, and performance. Consensus-based distributed control emerges as the leading approach to achieving fully distributed current sharing^9,15–17^.

Distributed control has gained significant attention in the literature, with remarkable advancement in its design and implementation aspects. For instance, Plug and Play (PnP) regulators have been employed to facilitate seamless integration and scalability^18,19^. Distributed control with event-triggered conditions has been proposed in^19,20^ to alleviate communication constraints while preserving system performance. In addressing cyber-resilience, several robust strategies against cyber-attacks were proposed to ensure operational security and reliability^21–23^.

Despite these advancements, which primarily aim to enhance robustness and reliability, the impact of sensor faults is often overlooked —a critical issue that can potentially jeopardize the reliability to be achieved.

Since voltage and current sensors are not ideal and might be subject to faults due to device aging and environmental changes, consequently, regardless of the control scheme employed (centralized, decentralized, or distributed), the controller may fail to provide a reliable and safe control command to the Microgrid when the sensors are biased, drifted, degraded in accuracy, or failed^24,25^. More importantly, considering the critical applications of DC Microgrids and their low impedances, protection systems are typically designed to be triggered within a few milliseconds. Several protection systems normally diagnose faults within 1 ms to 0.2 ms^26,27^ and relay on real-time accurate (assumed non-faulty) data from voltage and/or current sensors. Therefore, sensor faults can lead to incorrect triggering of the protection system, resulting in multiple faults, undermining the reliability of DC Microgrid operation, and affecting the stability of the overall grid. Therefore, automatically clearing faults within a short time is critical to ensure rapid detection and isolation while preserving system stability, and increasing the DC Microgrid reliability and resiliency. Recently, scholars have put forward a few methods to tolerate sensor faults in DC Microgrids. In^28^, the authors proposed a decentralized droop-based super-twisting sliding mode controller combined with three observers to tolerate sensor faults in DC Microgrids. However, despite using three observers for analytical redundancy, this scheme cannot handle simultaneous sensor faults. Additionally, the fault mitigation time is from 0.45s to 0.6s, which is slower considering the fast response of protection schemes; such delay may compromise the method’s effectiveness in critical applications. Another decentralized method proposed in^29^ is a power management scheme for DC Microgrid to maintain voltage stabilization in the presence of voltage sensor faults. However, this method does not tolerate sensor fault but a contingency algorithm in case of a DC-bus sensor failure or abnormality, focusing solely on the voltage sensor while ignoring the possible fault in the current sensor. A hybrid sensor fault tolerant control scheme was proposed for DC Microgrids in^30^ using extra sensors and a sliding mode observer. However, reliance on hardware redundancy introduces additional costs. Furthermore, hardware-based solutions do not guarantee complete resiliency. In^31^, a centralized predictive controller (MPC) coupled with a Dual-Extended Kalman Filter (DEKF) was utilized as a fault-tolerant control scheme for DC Microgrid. However, the combination of EKF and MPC introduces a substantial computational burden, making real-time execution impractical; this issue is exacerbated by the centralized nature of the proposed scheme, which increases computational and communication costs as the size of the DC Microgrid grows. Alternatively, a distributed H∞ observer scheme was proposed in^32^, where the observer depends on information exchanged through a communications network among DGUs. Although the distributed approach enhances the scalability relative to the centralized one, it is inadequate for sensor fault-tolerant control (FTC) in practical applications. Sensor FTC requires swift and autonomous fault mitigation, which cannot be guaranteed when depending on low-bandwidth communications. Furthermore, employing communications to accommodate sensor faults creates vulnerabilities due to their susceptibility to delay, noise, and cyber-attacks^28,33^.

Another notable limitation of the methods proposed in^28,30^ is their dependency on residual-based fault isolation techniques. Although effective in detecting predefined faults, these methods encounter difficulties in detecting and identifying unknown faults. Additionally, the residual signal frequently lacks persistence, making intermittent or time-varying faults difficult to identify due to the complication of setting appropriate thresholds for residual evaluation; stringent thresholds may lead to false positives, whereas lenient thresholds could neglect authentic faults, undermining system reliability.

Based on the above observations, this paper seeks to address the shortcomings of existing sensor fault-tolerant methodologies for DC Microgrids, including costly hardware redundancy, the complexity and the computational cost of deploying and employing multiple observers, dependence on communication networks for fault resilience, significant computational demands, accommodation of specific fault types, and delays in fault mitigation.

This paper proposes a real-time sensor fault identification and FTC scheme for DC Microgrids to address these challenges. The proposed approach employs a single Proportional Integral Unknown Input Observer (PI-UIO), markedly decreasing computational expenses and eliminating the necessity for costly redundant hardware. The observer provides real-time simultaneous current and voltage sensor fault estimation, which is utilized to reconfigure the Passivity-Based Voltage Controller (PBC) and the consensus current-sharing algorithm. The FTC scheme and the PBC are explicitly designed utilizing solely local DGU parameters and measurements, thereby circumventing dependence on communication networks. Thus, this improves the DC Microgrid reliability and scalability while also rendering it inherently resilient to communication constraints and cyber threats. The proposed scheme effectively addresses various sensor fault types, facilitating swift fault detection and resolution at both primary and secondary control levels. This method offers remarkable autonomy and real-time fault tolerance for DC Microgrids by operating as an intelligent local layer at each DGU. In summary, this paper provides the following contributions:

1. A single proportional-integral unknown input observer (PI-UIO) is employed to tolerate faults in both voltage and current sensors simultaneously. This is in contrast to previous works such as^28^, which relies on multiple observers, and^30^, which requires additional physical sensors to achieve fault tolerance.
2. The proposed framework achieves a fault accommodation time of 5 µs and a transient duration of only 5 ms due to controller reconfiguration. This is a significant improvement over methods like^28^, where the fault-tolerant response time is between 0.4 s and 0.6 s.
3. The proposed FTC operates autonomously without requiring communication between agents. In contrast to^32^, which depends on communication for FTC and is thus vulnerable to cyber-attacks, delays, and bandwidth limitations, the proposed method enhances resilience and responsiveness by eliminating these dependencies.
4. The proposed framework enables plug-and-play integration of DGUs even when local sensors are faulty or completely failed. This ensures seamless system reconfiguration and continued operation without requiring healthy sensors at the connecting unit.

This paper is organized as follows. In section II, the model of the adopted DC Microgrid is presented. Besides, some necessary preliminaries of graph theory and the sensor fault model are briefly established. The sensor fault tolerant control scheme with its proof is provided in Section III. In Section IV, the efficacy of the proposed approach is validated through real-time simulation in an OPAL-RT environment. Conclusion is give in Section V.

## Preliminaries

### Graph theory

**Fig. 1:**
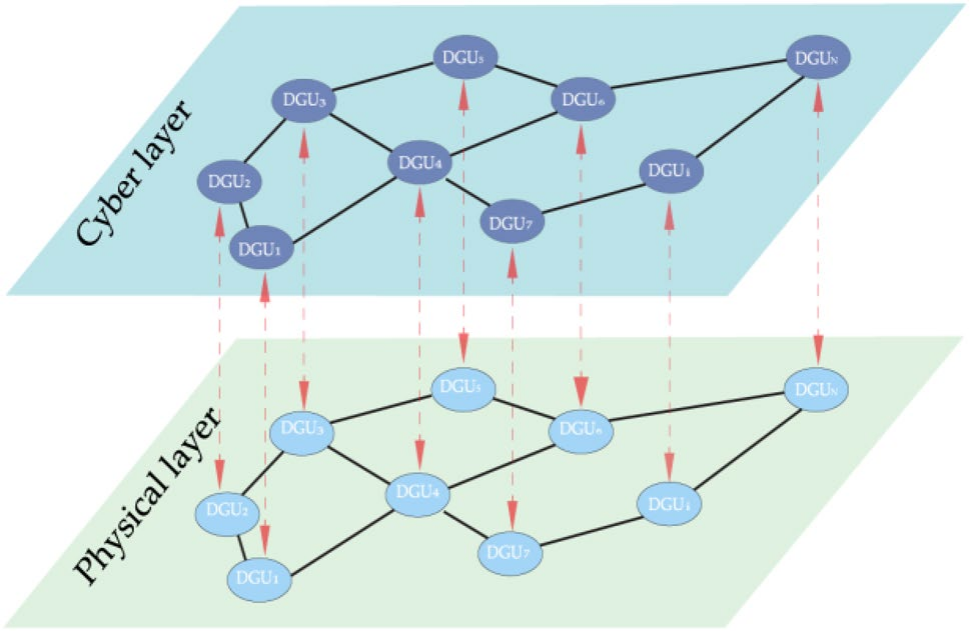
Communication and physical layers of n DGUs in a DC Microgrid.

In the distributed DC Microgrids, DGUs propagate data through a communication layer, which can be represented as undirected graph as $\:{\mathcal{G}}_{c}=(\mathcal{V},{\mathcal{E}}_{c},{\mathcal{W}}_{c})$, where $\:\mathcal{V}=\{{\mathcal{v}}_{1},{\mathcal{v}}_{2},{\mathcal{v}}_{3},..{,\mathcal{v}}_{N}\}$ is a finite set of *n* nodes i.e. set of DGUs and $\:{\mathcal{E}}_{c}\subseteq\:\mathcal{V}\times\:\mathcal{V}$ is a set of edges (communications links among DGUs) and $\:{\mathcal{W}}_{c}=\left[{a}_{ij}^{c}\right]\in\:{\mathbb{R}}^{n\times\:n}$ is the non-negative adjacency matrix of the graph $\:{\mathcal{G}}_{c}$, where $\:{a}_{ij}^{c}=1\:$if $\:{(\mathcal{v}}_{i},{\mathcal{v}}_{j})\in\:{\mathcal{E}}_{c}$ and $\:{a}_{ij}^{c}=0$ otherwise. The electrical network is modelled as weighted graph $\:{\mathcal{G}}_{e}=(\mathcal{V},{\mathcal{E}}_{e},{\mathcal{W}}_{e})$ where $\:\mathcal{V}=\{{\mathcal{v}}_{1},{\mathcal{v}}_{2},{\mathcal{v}}_{3},..{,\mathcal{v}}_{N}\}$ is a finite set of *n* DGUs and the edges $\:{\mathcal{E}}_{c}\subseteq\:\mathcal{V}\times\:\mathcal{V}$ represent the distribution lines interconnecting the DGUs. $\:{\mathcal{W}}_{e}=\left[{a}_{ij}^{e}\right]\in\:{\mathbb{R}}^{n\times\:n}$ represent the conductance matrix of the transmission lines where $\:{a}_{ij}^{e}=1/{R}_{ij}$*if* $\:{(\mathcal{v}}_{i},{\mathcal{v}}_{j})\in\:{\mathcal{E}}_{c}$ and $\:{a}_{ij}^{c}=0$ otherwise. The neighbour set of node *i* is denoted as$\:\:{\mathcal{N}}_{i}=\{{\mathcal{v}}_{i}:({\mathcal{v}}_{i},{\mathcal{v}}_{j})\in\:\mathcal{E}$^16,34^. The overall structure of the cyber (communication) and physical layers (electrical network) of the *n* DGUs is illustrated in Fig. 1.

### Model of the dc microgrid

**Fig. 2:**
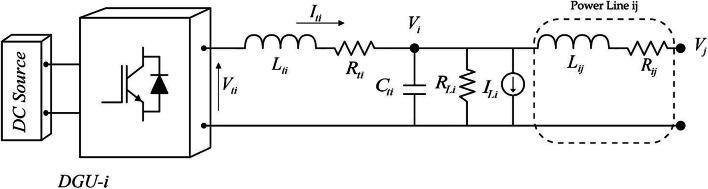
Electrical structure of a DGU.

In this study, a DC Microgrid consisting of $\:n$ DGUs connected by distribution lines is considered. Typically, each DGU comprises a primary DC energy source, a DC-DC converter, and local loads. Figure 2 illustrates the block diagram of a typical converter-based distributed generation electrical configuration.

Assuming that the power lines connecting DGUs have quasi-stationary dynamics^19,35^ and based on Kirchhoff’s voltage law (KVL), the following dynamic model is obtained for the *i*^th^ distribution line:

1 $$\:DGUi\::\:\left\{\begin{array}{c}\frac{dVi}{dt}=\frac{1}{{C}_{ti}}{I}_{ti}-\frac{1}{{C}_{ti}}{I}_{{L}_{i}}-\frac{1}{{{C}_{ti}R}_{Li}}{V}_{i}+\sum\:_{j\in\:{\mathcal{N}}_{i}}\frac{{V}_{j}-{V}_{i}}{{{C}_{ti}R}_{ij}}\\\:\frac{d{I}_{ti}}{dt}=-\frac{1}{{L}_{ti}}{V}_{i}-\frac{{R}_{ti}}{{L}_{ti}}{I}_{ti}+\frac{1}{{L}_{ti}}{V}_{ti}\:\:\:\:\:\:\:\:\:\:\:\:\:\:\:\:\:\:\:\:\:\:\:\:\:\:\:\:\end{array}\right.$$

where $\:{V}_{i}$ and $\:{I}_{ti}$ are the voltage and the current at the common coupling point (PCC) and the output current of DGU-*i*, respectively. $\:{V}_{ti}$ is the output voltage of the power converter *i*, $\:{I}_{{L}_{i}}$ is the load current, $\:{L}_{ti}$ and $\:{C}_{ti}$ are the inductance and the capacitance of DGU-*i*, respectively. $\:{V}_{j}$ is the voltage at the PCC of each neighbouring DGU-*j* $\:j\in\:{\mathcal{N}}_{i,}$ and the conductance of the power line connecting the DGU-*i* to its neighboring DGU-*j* is denoted by $\:\frac{1}{{R}_{ij}}$. A primary decentralized controller is designed to regulate the voltage at each PCC and ensure a stable Microgrid. The DGUs can supply uninterrupted loads only when the proportional current sharing among the DGUs is ensured. The consensus algorithms are usually employed to reach an agreement upon a certain quantity of interests in multi-agent systems^15,16,36^. In this paper, the consensus-based cooperative control is adopted. Thus, a secondary controller is designed for the DGU-*i* as follows:

2 $$\:{\dot{{\Delta\:}V}}_{i}=-{\sum\:}_{j\in\:{\mathcal{N}}_{i}}^{\:}{a}_{ij}^{c}\left(\frac{{I}_{ti}}{{I}_{ti}^{p}}-\frac{{I}_{tj}}{{I}_{tj}^{p}}\right)$$

where $\:{I}_{ti}^{p}$ are constant scaling factors set to the corresponding DG-rated current to make DGUs share the total load power proportionally with their generation capacities. From a system point of view, the collective dynamics of the group of DGUs following (2) can be expressed as:

3 $$\:\dot{\varDelta\:V}=-\mathbb{L}\mathbb{D}{I}_{t}$$

where $\:\dot{\varDelta\:V}=\left[\varDelta\:{V}_{1},...,\varDelta\:{V}_{N}\right]$, $\:{I}_{t}=\left[{I}_{t1},\dots\:,{I}_{tN}\right]$, $\:\mathbb{D}=diag\left(\frac{1}{{I}_{t1}},\dots\:,\frac{1}{{I}_{tN}}\right)$, and $\:\mathbb{L}$ is the Laplacian matrix of $\:{\mathcal{G}}_{c}$. Since the communication network holds the same topology as the electrical network, the coefficients in Eq. (2) are set as $\:{a}_{ij}^{c}=\lambda\:/{R}_{ij}$ so that the current sharing at steady state reaches a stable consensus. For a thorough mathematical theoretical proof, the consensus-based algorithm can be referenced in^16^.

### Problem formulation

The voltage and current sensors are crucial parts of the DC Microgrid as they are both used as feedback for the control system and, in many cases, for the protection system. Therefore, erroneous sensor measurements could lead to reduced system performance, instability, damage of components, and incorrect triggering of the protection system due to the low impedance of the DC Microgrid fault protection schemes, such as fuses and relays that are usually triggered within a few milliseconds. Sensors are employed to provide feedback on the required output voltage and inductor current information for the control system. However, in practice, sensor faults/failures are prone to occur, either due to a single event or to accumulated degradation over time, classified as catastrophic and wear-out failures exhibiting the following faults modes: -Complete sensor failures - DC bias in sensor measurement - Time-varying disturbances. Generally, these faults/failures can be mathematically modeled as affine deviations in the output as in (4)^37,38^:

4 $$\:{y}^{f}\left(t\right)=\stackrel{-}{C}{x}_{i}\left(t\right)+\stackrel{-}{D}{f}_{si}\left(t\right)$$

where $\:\stackrel{-}{D}$ is an identity distribution matrix, $\:{f}_{si}\left(t\right)$ represents the unknown sensor fault signal in the corresponding sensor.

As clearly manifested in Eq. (1), the dynamics of the DC Microgrid are influenced by the information exchange and parameters of the other DGUs. More specifically, the term $\:\sum\:_{j\in\:{\mathcal{N}}_{i}}\frac{{V}_{j}-{V}_{i}}{{{C}_{ti}R}_{ij}}$ describes the interactions between DGU-*i* and its neighboring connections, which are usually shared through communications networks. However, practical applications often require data transmission as less as possible due to the limitation of communication bandwidth. Additionally, uncertainties in the network, external disturbance, delays, cyber-attacks, and noisy environment can further influence the fault accommodations.

The primary objective of this paper is to design a scalable sensor fault-tolerant control scheme capable of ensuring system resiliency in the presence of sensor faults without relying on communications.

### Sensor fault-tolerant control scheme

In this section, an observer-based FTC scheme will be designed for resilient voltage regulation and current sharing under sensor faults. First, a PI-UIO is leveraged to estimate the sensor faults in both states (PCC voltage and current) of an islanded DC Microgrid of *n* DGUs without reliance on communications. Then, a decentralized PBC controller is constructed to guarantee the stability of the voltage reference tracking system. Furthermore, the control reconfiguration will be presented to achieve fault accommodations in the presence of sensor faults.

### Design of proportional integral unknown input observer

The coupling terms $\:\sum\:_{j\in\:{\mathcal{N}}_{i}}\frac{{V}_{j}-{V}_{i}}{{R}_{ij}}$ in (1) not only limit the scalability but also require communications, which could make the control system and/or FTC scheme vulnerable to malicious attacks, delay, high cost, etc. Additionally, it constrains the PnP operation since all DGUs will need to be updated when some DGUs are plugged out or in. Therefore, in order to eliminate the need for communications, the coupling terms are considered as unknown disturbances to DGU-*i.* Thus, the dynamics of DGU-*i* given in (1) can be expressed as: 

Where $${x_i} = \left[ {\begin{array}{*{20}{c}} {{x_1}}&{{x_2}} \end{array}} \right] = \left[ {\begin{array}{*{20}{c}} {{V_i}}&{{I_{ti}}} \end{array}} \right]$$ $${u_i} = \left[ {\begin{array}{*{20}{c}} {{I_{{L_i}}}}&{{V_{ti}}} \end{array}} \right]$$ $$\,{\xi _i}\left( t \right) = \mathop \sum \limits_{j \in {\mathcal{N}_i}} \frac{{{V_j} - {V_i}}}{{{R_{ij}}}}$$ 

5 $$\:{\dot{x}}_{i}\left(t\right)={\stackrel{-}{A}}_{ii}{x}_{i}\left(t\right)+{\stackrel{-}{B}}_{i}{u}_{i}\left(t\right)+{\stackrel{-}{E}}_{i}{\xi\:}_{i}\left(t\right)$$

6 $$\:{y}_{i}\left(t\right)=\stackrel{-}{C}{x}_{i}\left(t\right)$$

$$\:{\stackrel{-}{A}}_{ii}=\left[\begin{array}{cc}-\frac{1}{{{C}_{ti}R}_{Li}}&\:\frac{1}{{C}_{ti}}\\\:-\frac{1}{{L}_{ti}}&\:-\frac{{R}_{ti}}{{L}_{ti}}\end{array}\right];\:{\stackrel{-}{B}}_{i}=\left[\begin{array}{cc}-\frac{1}{{C}_{ti}}&\:0\\\:0&\:-\frac{1}{{L}_{ti}}\end{array}\right];\:{\stackrel{-}{E}}_{i}=\left[\begin{array}{c}\frac{1}{{C}_{ti}}\\\:0\end{array}\right];\:\stackrel{-}{C}=\left[\begin{array}{cc}1&\:0\\\:0&\:1\end{array}\right]\:;$$.

Considering the sensor fault in (4) and using Eq. (6), the DGU-*i* model considering a sensor fault can be expressed as follows:

7 $$\:{\dot{z}}_{i}\left(t\right)={A}_{ii}{z}_{i}\left(t\right)+{B}_{i}{u}_{i}\left(t\right)+{E}_{i}{\xi\:}_{i}\left(t\right)$$

8 $$\:{y}_{i}\left(t\right)=C{z}_{i}\left(t\right)$$

where $$\:{z}_{i}=\left[\begin{array}{cc}{x}_{i}&\:{f}_{si}\end{array}\right]$$

$$\:{A}_{ii}=\left[\begin{array}{cc}A&\:D\\\:0&\:0\end{array}\right];\:{B}_{i}=\left[\begin{array}{c}{\stackrel{-}{B}}_{i}\\\:0\end{array}\right];{E}_{i}=\left[\begin{array}{c}{\stackrel{-}{E}}_{i}\\\:0\end{array}\right];\:C=\left[\begin{array}{cc}\stackrel{-}{C}&\:0\end{array}\right]$$.

An observer for each DGU-*i* is constructed as:

9 $$\:\begin{array}{c}\dot{{\zeta\:}_{i}}\left(t\right)={\mathcal{F}}_{ii}{\zeta\:}_{i}\left(t\right)+{\mathcal{J}}_{i}{{B}_{i}u}_{i}\left(t\right)+{\mathcal{H}}_{i}{y}_{i}\left(t\right)\\\:\widehat{z}\left(t\right)={\zeta\:}_{i}\left(t\right)+{\mathcal{T}}_{i}{y}_{i}\left(t\right)\:\:\:\:\:\:\:\:\:\:\:\:\:\:\:\:\:\:\:\:\:\:\:\:\:\:\:\:\:\:\end{array}$$

where $$\:\widehat{z}\left(t\right)=\left[\begin{array}{cc}{\widehat{x}}_{i}&\:{\widehat{f}}_{si}\end{array}\right]$$ is the augmented state estimate, and $\:{\mathcal{F}}_{ii}$, $\:{\mathcal{J}}_{i}$,$\:{\mathcal{\:}\mathcal{H}}_{i},and\:{\mathcal{T}}_{i}\:$are design matrices to be determined. Defining the state estimation error as $\:{e}_{i}\left(t\right)={z}_{i}\left(t\right)-{\widehat{z}}_{i}\left(t\right),$ which can be expressed as:

10 $$\:{e}_{i}\left(t\right)=\:{z}_{i}\left(t\right)-{\zeta\:}_{i}\left(t\right)-{\mathcal{T}}_{i}{y}_{i}\left(t\right)\:=\left(I-{\mathcal{T}}_{i}C\right){z}_{i}\left(t\right)-{\zeta\:}_{i}\left(t\right)$$

Thus, the estimation error dynamics are expressed as:

$$\:{\dot{e}}_{i}\left(t\right)\:=\left(I-{\mathcal{T}}_{i}C\right){\dot{z}}_{i}\left(t\right)-{\dot{\zeta\:}}_{i}\left(t\right)$$

$$\:{\dot{e}}_{i}\left(t\right)\:=\left(I-{\mathcal{T}}_{i}C\right)\left({A}_{ii}{z}_{i}\left(t\right)+{B}_{i}{u}_{i}\left(t\right)+{E}_{i}{\xi\:}_{i}\left(t\right)\right)-{\mathcal{F}}_{ii}{\zeta\:}_{i}\left(t\right)-{\mathcal{J}}_{i}{{B}_{i}u}_{i}\left(t\right)-{\mathcal{H}}_{i}{y}_{i}\left(t\right)$$

$$\:{\dot{e}}_{i}\left(t\right)\:={A}_{ii}{z}_{i}\left(t\right)+{B}_{i}{u}_{i}\left(t\right)+{E}_{i}{\xi\:}_{i}\left(t\right)-{\mathcal{T}}_{i}C{A}_{ii}{z}_{i}\left(t\right)-{\mathcal{T}}_{i}C{B}_{i}{u}_{i}\left(t\right)\:\:-{\mathcal{T}}_{i}C{E}_{i}{\xi\:}_{i}\left(t\right)-{\mathcal{F}}_{ii}{\zeta\:}_{i}\left(t\right)-{\mathcal{J}}_{i}{{B}_{i}u}_{i}\left(t\right)-{\mathcal{H}}_{i}C{z}_{i}\left(t\right)$$

$$\:{\dot{e}}_{i}\left(t\right)=\left({A}_{ii}-{{\mathcal{T}}_{i}CA}_{ii}-{\mathcal{H}}_{i}C\right){z}_{i}+\left({B}_{i}-{\mathcal{T}}_{i}C{B}_{i}-{\mathcal{J}}_{i}{B}_{i}\right){u}_{i}\left(t\right)\:+\:\left({E}_{i}-{\mathcal{T}}_{i}C{E}_{i}\right){\xi\:}_{i}\left(t\right)\:-{\mathcal{F}}_{ii}\widehat{z}\left(t\right)-{\mathcal{F}}_{ii}{\mathcal{T}}_{i}C{z}_{i}\left(t\right)$$

11 $$\:{\dot{e}}_{i}\left(t\right)\:=\left({A}_{ii}-{{\mathcal{T}}_{i}CA}_{ii}-{\mathcal{K}}_{i}C-{\mathcal{F}}_{ii}\right){z}_{i}\left(t\right)+({B}_{i}-{\mathcal{T}}_{i}C{B}_{i}-{\mathcal{J}}_{i}{B}_{i}){u}_{i}\left(t\right)+\:\left({E}_{i}-{\mathcal{T}}_{i}C{E}_{i}\right){\xi\:}_{i}\left(t\right)+{\mathcal{F}}_{ii}{e}_{i}\left(t\right)$$

$$\:\mathrm{w}\mathrm{h}\mathrm{e}\mathrm{r}\mathrm{e}\:{\mathcal{L}}_{i}={\mathcal{F}}_{ii}{\mathcal{T}}_{i},\:\mathrm{a}\mathrm{n}\mathrm{d}\:{\mathcal{K}}_{i}={\mathcal{H}}_{i}-{\mathcal{L}}_{i}.$$.

If one can satisfy the following conditions:

12 $$\:\left(I-{\mathcal{T}}_{i}C\right){E}_{i}=0$$

13 $$\:{\mathcal{J}}_{i}=I-{\mathcal{T}}_{i}C$$

14 $$\:{\mathcal{F}}_{ii}={A}_{ii}-{{\mathcal{T}}_{i}CA}_{ii}-{\mathcal{K}}_{i}C={A}_{\mathcal{O}ii}-{\mathcal{K}}_{i}C$$

15 $$\:{\mathcal{K}}_{i}={\mathcal{H}}_{i}-{\mathcal{L}}_{i}$$

Condition (12) plays a fundamental role in the proposed observer design. Specifically, the term $\:{\mathrm{E}}_{\mathrm{i}}{{\upxi\:}}_{\mathrm{i}}\left(\mathrm{t}\right)$ captures the coupling effects arising from neighboring DGUs and network interconnections. By enforcing $\:\left(\mathrm{I}-{\mathcal{T}}_{\mathrm{i}}\mathrm{C}\right){\mathrm{E}}_{\mathrm{i}}=0$ the influence of this coupling term is completely eliminated from the observer error dynamics. As a result, the estimation error evolution becomes fully decoupled from neighboring units and depends only on local variables. This property is essential to guarantee decentralized operation and enables plug-and-play functionality without requiring parameter reconfiguration or communication among DGUs. Consequently, the state estimation error of each DGU-i is rearranged to be:

16 $$\:{\dot{e}}_{i}\left(t\right)={\mathcal{F}}_{ii}{e}_{i}\left(t\right)$$

If all eigenvalues of $\:{\mathcal{F}}_{ii}$ are stable, $\:\:{e}_{i}\left(t\right)$ will approach zero asymptotically, i.e. $\:{\widehat{f}}_{si}$→ $\:{\widehat{f}}_{si}$. The sufficient conditions to ensure the stabilization of $\:{\mathcal{F}}_{ii}$ are as follows:

i) Rank(C*E*_*i*_)=rank(*E*_*i*_).
ii) (*C*,$\:{A}_{\mathcal{O}ii}$) is a detectable pair.

The observer design involves solving (12)-(15) while placing all the eigenvalues of the system matrix $\:{\mathcal{F}}_{ii}$ on the left half of the complex plane. Consequently, the UI-PIO expressed in (9) can be used to estimate the sensor faults that will be used later in the fault-tolerant process completely independent of the neighboring DGUs, hence no reliance on communications, which could enhance the fault mitigation time. The error dynamics (16) also have no interconnection terms among DGUs. However, switch-mode power converters are subjected to noise. It has been shown in^39,40^ that measurement noise can have the most dominant influence on the converter’s performance; therefore, suppressing the measurement noise on the fault estimation can further enhance the accuracy of the fault accommodation. In the proposed observer, the sensor noise is taken into account, and the _∞_ optimization is applied to minimize the influence of the sensor noise on the fault estimation signal. When the output measurement is corrupted by sensor noise (8), it is written as:

17 $$\:{y}_{i}\left(t\right)=C{z}_{i}\left(t\right)+{F}_{s}{d}_{s}\left(t\right)$$

Using (7) and taking the noise (17) into consideration, the estimation error is as follows:

18 $$\:{\dot{e}}_{i}\left(t\right)={\mathcal{F}}_{ii}{e}_{i}\left(t\right)-\:{\mathcal{K}}_{i}{F}_{s}{d}_{s}\left(t\right)-{\mathcal{T}}_{i}{F}_{s}{\dot{d}}_{s}\left(t\right)$$

The measurement noise affects the estimation error. In the following, the $\:{\mathcal{H}}_{\infty\:}$ performance index is imposed to minimize the effect of noise on the fault estimation, which is defined as:

19 $$\:{\left\| \mathcal{H} \right\|_{\infty \:}} = \mathop {sup}\limits_{\left\| {\Delta \psi } \right\|{\:_{{\mathcal{L}_2}}} \ne \:0} \frac{{{{\left\| {{r_i}\left( t \right)} \right\|}_{{\mathcal{L}_2}}}}}{{\left\| {\Delta \psi \:\left( t \right)} \right\|{\:_{{\mathcal{L}_2}}}}} \leqslant \:\sqrt {\sigma \:}$$

where $$\:\varDelta\:\psi\:=\left[\begin{array}{cc}{d}_{s}&\:{\dot{\dot{d}}}_{s}\end{array}\right]$$ and $\:{r}_{i}\left(t\right)={y}_{i}\left(t\right)-C\widehat{z}\left(t\right)=C{e}_{i}\left(t\right)+{F}_{s}{d}_{s}$. To ensure stability, the following Lyapunov function is considered:

20 $$\:{W}_{i}\left({e}_{i}\right)={e}_{i}^{T}{P}_{i}{e}_{i}$$

Using the error dynamics given in (18), the time derivative of $\:{W}_{i}$ becomes:

$$\:{\dot{W}}_{i}={\dot{e}}_{i}^{T}{P}_{i}{e}_{i}+{e}_{i}^{T}{P}_{i}{\dot{e}}_{i}$$

$$\:{\:\:\:\:\:\:{\dot{W}}_{i}\:=\left({\mathcal{F}}_{ii}{e}_{i}\left(t\right)-\:{\mathcal{K}}_{i}{F}_{s}{d}_{s}-{\mathcal{T}}_{i}{F}_{s}{\dot{d}}_{s}\right)}^{T}{P}_{i}{e}_{i}\:\:+{e}_{i}^{T}{P}_{i}\left({\mathcal{F}}_{ii}{e}_{i}\left(t\right)-\:{\mathcal{K}}_{i}{F}_{s}{d}_{s}-{\mathcal{T}}_{i}{F}_{s}{\dot{d}}_{s}\right)$$

21 $$\:\:\:\:\:\:{\dot{W}}_{i}\:\:={e}_{i}^{T}\left({P}_{i}\left({A}_{\mathcal{O}ii}-{\mathcal{K}}_{i}C\right)+{\left({A}_{\mathcal{O}ii}-{\mathcal{K}}_{i}C\right)}^{T}{P}_{i}\right){e}_{i}-2{e}_{i}{P}_{i}{\mathcal{K}}_{i}{F}_{s}{d}_{s}-2{e}_{i}{P}_{i}{\mathcal{T}}_{i}{F}_{s}{\dot{d}}_{s}\:$$

To minimize the effect of measurement noise on the estimation in the $\:{\mathcal{L}}_{2}$ sense, the following constraint on the stability criteria is imposed:

22 $$\:J\left(t\right)={\dot{W}}_{i}\left({e}_{i}\right)+{r}^{T}\left(t\right)r\left(t\right)-\sigma\:\varDelta\:{\psi\:}^{T}\left(t\right)\varDelta\:\psi\:\left(t\right)\le\:0$$

Integrating both sides of (22) with respect to *t* over the time period $\:\left[0,\infty\:\right]$ gives:

23 $$\:{\:\:\:\:\:\:\:W}_{i}\left({\infty\:}\right)-{W}_{i}\left(0\right)+{\int\:}_{0}^{{\infty\:}}\left({r}^{T}\left(t\right)r\left(t\right)-\sigma\:\varDelta\:{\psi\:}^{T}\left(t\right)\varDelta\:\psi\:\left(t\right)\right)dt\le\:0$$

As $\:{W}_{i}\left(\infty\:\right)\ge\:0$ and under zero initial conditions $\:{W}_{i}\left(0\right)=0$ one obtains:

24 $$\:\:\:\:\:\:\:\:\:\sqrt{{\int\:}_{0}^{{\infty\:}}{r}^{T}\left(t\right)r\left(t\right)dt}\le\:\sqrt{\sigma\:}\sqrt{{\int\:}_{0}^{{\infty\:}}\varDelta\:{\psi\:}^{T}\left(t\right)\varDelta\:\psi\:\left(t\right)dt}$$

Therefore, it results:

25 $$\:\:\:\:\:\:\:\:\:\:\:\:\:\:\:\:\:\frac{\sqrt{{\int\:}_{0}^{{\infty\:}}{r}^{T}\left(t\right)r\left(t\right)dt}}{\sqrt{{\int\:}_{0}^{{\infty\:}}\varDelta\:{\psi\:}^{T}\left(t\right)\varDelta\:\psi\:\left(t\right)dt}}\le\:\sqrt{\sigma\:}$$

Equivalently $\:{\left\| \mathcal{H} \right\|_{\infty \:}} \leqslant \:0,\:i.e.,$ if (22) held true/negative, it enforces the minimization of the effect of measurement noise on the estimation error that is the same as the condition in (25). From (21) and after some algebraic manipulation of (22) and using the definition of $\:{r}_{i}$ we obtain:

26 $$\:J\left(t\right)={e}_{i}^{T}\left({P}_{i}\left({A}_{\mathcal{O}ii}-{\mathcal{K}}_{i}C\right)+{\left({A}_{\mathcal{O}ii}-{\mathcal{K}}_{i}C\right)}^{T}{P}_{i}\right){e}_{i}-\:\:2{e}_{i}^{T}{P}_{i}{\mathcal{K}}_{i}{F}_{s}{d}_{s}-2{e}_{i}^{T}{P}_{i}{\mathcal{T}}_{i}{F}_{s}{\dot{d}}_{s}+{e}_{i}^{T}{C}^{T}C{e}_{i}+{e}_{i}^{T}{C}^{T}{F}_{s}{d}_{s}+{d}_{s}^{T}{F}_{s}^{T}C{e}_{i}+{d}_{s}^{T}{F}_{s}^{T}{F}_{s}{d}_{s}-\sigma\:\varDelta\:{\psi\:}^{T}\varDelta\:\psi\:\le\:0$$

Equivalent to:

27 $$\:J\left(t\right)\le\:{\left[\begin{array}{ccc}{e}_{i}&\:{d}_{s}&\:{\dot{d}}_{s}\end{array}\right]}^{T}\left(\left[\begin{array}{ccc}{P}_{i}\left({A}_{\mathcal{O}ii}-{\mathcal{K}}_{i}C\right)+{\left({A}_{\mathcal{O}ii}-{\mathcal{K}}_{i}C\right)}^{T}{P}_{i}&\:{-P}_{i}{\mathcal{K}}_{i}{F}_{s}&\:{-P}_{i}{\mathcal{T}}_{i}{F}_{s}\\\:\mathrm{*}&\:-\sigma\:I&\:0\\\:\mathrm{*}&\:\mathrm{*}&\:-\sigma\:I\end{array}\right]+\:\:\:\:\:\:\left[\begin{array}{ccc}{C}^{T}C&\:{C}^{T}{F}_{s}&\:0\\\:\mathrm{*}&\:{F}_{s}^{T}{F}_{s}&\:0\\\:\mathrm{*}&\:\mathrm{*}&\:0\end{array}\right]\right)\left[\begin{array}{c}{e}_{i}\\\:{d}_{s}\\\:{\dot{d}}_{s}\end{array}\right]$$

Using Schur-compliment, one obtains:

28 $$\:j\left(t\right)\le\:{\chi\:}_{i}^{T}{{\Sigma\:}}_{i}{\chi\:}_{i}\le\:0$$

where $$\:{\chi\:}_{i}^{}={\left[\begin{array}{cc}{e}_{i}&\:\varDelta\:\psi\:\end{array}\right]}^{T}$$

29 $$\:{\varSigma\:}_{i}=\left[\begin{array}{ccc}{\varXi\:}_{ii}&\:{-Q}_{i}{F}_{s}&\:\begin{array}{cc}{-P}_{i}{\mathcal{T}}_{i}{F}_{s}&\:{C}^{T}\end{array}\\\:\mathrm{*}&\:-\sigma\:I&\:\begin{array}{cc}0&\:{F}_{s}^{T}\end{array}\\\:\begin{array}{c}\mathrm{*}\\\:\mathrm{*}\end{array}&\:\begin{array}{c}\mathrm{*}\\\:\mathrm{*}\end{array}&\:\begin{array}{cc}-\sigma\:I&\:0\\\:\mathrm{*}&\:-I\end{array}\end{array}\right]$$

$$\:\mathrm{w}\mathrm{i}\mathrm{t}\mathrm{h}\:{{\Xi\:}}_{ii}={P}_{i}{A}_{\mathcal{O}ii}-{Q}_{i}C+{A}_{\mathcal{O}ii}^{T}{P}_{i}-{C}^{T}{Q}_{i}\:{Q}_{i}={P}_{i}{\mathcal{K}}_{i}$$.

A sufficient condition for $\:J<0$ is $\:{\varSigma\:}_{i}\le\:0$. Thus, the observer error dynamics are stable with the prescribed $\:{\mathcal{H}}_{\infty\:}\:$attenuation level $\:\sqrt{\sigma\:}$. After solving the LMI (29), the gain $\:{\mathcal{K}}_{i}$ can be computed from$\:\:{\mathcal{K}}_{i}={P}_{i}^{-1}{Q}_{i}$. The stability analysis of the PI-UIO for fault estimation for each DGU can be derived similarly. It is worth noting that the observer only requires knowledge of the DGU-*i* local parameters and measurement, thus allowing for a scalable and modular design. The design procedure of the PI-UIO for sensor fault estimation can be summarized in the following steps:

1. *Construct the augmented system in the form of (7).*
2. *Solve (12) for* $\:{\mathcal{T}}_{i}$, *a particular solution is* $\:{\mathcal{T}}_{i}={E}_{i}{\left(C{E}_{i}\right)}^{+}$,*then solve (13) for* $\:{\mathcal{J}}_{i}$.
3. *Solve the LMI (29) to obtain* $\:{\mathcal{K}}_{i}$, *hence* $\:{\mathcal{F}}_{ii}$ *from (14) and* $\:{\mathcal{L}}_{i}$ *from (15).*
4. *Implement the augmented observer as in (9) for each DGU-i*,* and obtain the fault estimation*: $$\:{\widehat{f}}_{si}=\left[\begin{array}{cc}{0}_{n}&\:I\end{array}\right]\widehat{z}\left(t\right)$$.

It is worth noting that the LMI (29) defines a convex feasible set, which may admit multiple admissible solutions for$\:{P}_{i}$ and $\:{Q}_{i}$. In this work, LMI (29) is solved using the MATLAB YALMIP toolbox with a pre-specified attenuation level $\:\sqrt{\sigma\:}$.The value of $\:\sqrt{\sigma\:}$ is then iteratively adjusted to obtain a solution that is feasible while achieving a minimized attenuation level.

### Passivity-based controller

This section introduces a decentralized passivity-based controller (PBC) for voltage regulation designed to guarantee voltage stability in the DC Microgrid. The aim is to design a PBC voltage controller for each DGU-*i* individually without considering the interactions among different DGs.

Defining:

$$\:{\mathcal{Z}}_{i}=\left(\begin{array}{c}{V}_{i}\\\:{I}_{ti}\end{array}\right),\:{\mathcal{H}}_{i}=\left(\begin{array}{cc}{C}_{ti}&\:0\\\:0&\:{L}_{ti}\end{array}\right),\:\mathcal{G}=\left(\begin{array}{cc}0&\:-1\\\:1&\:0\end{array}\right),{\mathcal{\:}\mathcal{R}}_{i}=\left(\begin{array}{cc}\frac{1}{{R}_{Li}}&\:0\\\:0&\:0\end{array}\right),{\:{\Gamma\:}}_{\mathrm{i}\mathrm{j}}=\left(\begin{array}{c}\sum\:_{j\in\:{\mathcal{N}}_{i}}\frac{{V}_{j}-{V}_{i}}{{R}_{ij}}-{I}_{Li}\\\:{V}_{ti}\end{array}\right)$$

Then Eq. (1) can be rewritten in the following form:

30 $$\:{\mathcal{H}}_{\mathrm{i}}{\dot{\mathcal{Z}}}_{\mathrm{i}}+\left[\mathcal{G}+{\mathcal{R}}_{i}\right]{\mathcal{Z}}_{\mathrm{i}}={{\Gamma\:}}_{\mathrm{i}\mathrm{j}}$$

The objective of the controller is to maintain the voltage at the PCC at its constant desired value$\:\:{V}_{i}^{d}$. Thus, the error dynamics are obtained by reshaping the coordinates of (30) to$\:\:{\mathcal{Z}}_{i}={\stackrel{\sim}{\mathcal{Z}}}_{i}+{\mathcal{Z}}_{di}$, which yields:

31 $$\:{\mathcal{H}}_{i}{\dot{\stackrel{\sim}{\mathcal{Z}}}}_{i}+\left[\mathcal{G}+{\mathcal{R}}_{i}\right]{\stackrel{\sim}{\mathcal{Z}}}_{i}={\varGamma\:}_{ij}-\left({\mathcal{H}}_{i}\dot{{\mathcal{Z}}_{di}}+\left[\mathcal{G}+{\mathcal{R}}_{i}\right]{\mathcal{Z}}_{di}\right)$$

where $\:{\stackrel{\sim}{\mathcal{Z}}}_{i}$ is the deviation from the set point $\:{\mathcal{Z}}_{di}$.

By adding the desired damping injection resistance matrix $\:{\mathcal{R}}_{di}{\stackrel{\sim}{\mathcal{Z}}}_{i}$ for both sides of (31), the error dynamics are rewritten as:

32 $$\:{\mathcal{H}}_{i}{\dot{\stackrel{\sim}{\mathcal{Z}}}}_{i}+\left[\mathcal{G}+{\mathcal{R}}_{i}\right]{\stackrel{\sim}{\mathcal{Z}}}_{i}={\varGamma\:}_{ij}-\left({\mathcal{H}}_{i}\dot{{\mathcal{Z}}_{di}}+\left[\mathcal{G}+\mathcal{\:}{\stackrel{-}{\mathcal{R}}}_{i}\right]{\mathcal{Z}}_{di}-{\mathcal{R}}_{di}{\stackrel{\sim}{\mathcal{Z}}}_{i}\right)$$

*here*:

33 $$\:{\stackrel{-}{\mathcal{R}}}_{i}=\left(\begin{array}{cc}\frac{1}{{R}_{Li}}+\frac{1}{{R}_{d1}}&\:0\\\:0&\:{R}_{d2}\end{array}\right),{\mathcal{R}}_{d}={\stackrel{-}{\mathcal{R}}}_{i}-{\mathcal{R}}_{i}=\left(\begin{array}{cc}\frac{1}{{R}_{d1}}&\:0\\\:0&\:{R}_{d2}\end{array}\right)$$

Due to the addition of virtual resistance, which guarantees the dissipation of transient energy of the system and also concurs with the Lyapunov sense, the left-hand side of (32) reaches a globally asymptotically stable equilibrium point^6,41^. Let consider the Lyapunov function $\:{\mathcal{V}}_{i}\left({\stackrel{\sim}{\mathcal{Z}}}_{i}\right)=\frac{1}{2}{\stackrel{\sim}{\mathcal{Z}}}_{i}^{T}{\mathcal{H}}_{i}{\stackrel{\sim}{\mathcal{Z}}}_{i},$ where $\:{\mathcal{H}}_{i}\:$is a positive definite matrix. Taking the time derivative along the left-hand side of (32) trajectories yields $\:\dot{{\mathcal{V}}_{i}}\left({\stackrel{\sim}{\mathcal{Z}}}_{i}\right)=-{\stackrel{\sim}{\mathcal{Z}}}_{i}^{T}{\stackrel{-}{\mathcal{R}}}_{i}{\stackrel{\sim}{\mathcal{Z}}}_{i}<0$, since $\:\mathcal{G}$ skew symmetric matrix ($\:\mathcal{G}+{\mathcal{G}}^{T}=0)$ and $\:{\stackrel{-}{\mathcal{R}}}_{i}$ is positive definite. Hence, the equilibrium $\:{\stackrel{\sim}{\mathcal{Z}}}_{i}=0$, is globally asymptotically stable. Thus, the stable controller design can be carried out by identifying (32) as follows:

34 $$\:{\varGamma\:}_{ij}-\left({\mathcal{H}}_{i}\dot{{\mathcal{Z}}_{di}}+\left[\mathcal{G}+\mathcal{\:}{\stackrel{-}{\mathcal{R}}}_{i}\right]{\mathcal{Z}}_{di}-{\mathcal{R}}_{di}{\stackrel{\sim}{\mathcal{Z}}}_{i}\right)=0$$

Thus, using the definition of $\:{\varGamma\:}_{ij},$ $\:{\mathcal{H}}_{i}$ $\:\mathcal{G},{\mathcal{R}}_{di},and\:{\stackrel{-}{\mathcal{R}}}_{i}$, the Eq. (34) will be:

35 $$\:\:\:\:\:\:\:\:\:\:\:\sum\:_{j\in\:{\mathcal{N}}_{i}}\frac{{V}_{j}-{V}_{i}}{{R}_{ij}}-{I}_{Li}-{C}_{ti}\dot{{V}_{i}^{d}}+{I}_{ti}^{d}-\frac{{V}_{i}^{d}}{{R}_{Li}}+\frac{1}{{R}_{d1}}\left({V}_{i}-{V}_{i}^{d}\right)=0$$

36 $$\:{V}_{ti}-{L}_{ti}{\dot{I}}_{ti}^{d}-{V}_{i}-{R}_{d2}\left({I}_{ti}-{I}_{ti}^{d}\right)=0$$

Where $$\:{\stackrel{\sim}{\mathcal{Z}}}_{i}=\left[\begin{array}{c}{V}_{i}-{V}_{i}^{d}\\\:{I}_{ti}-{I}_{ti}^{d}\end{array}\right]$$.

Finally, from (35) and (36), the output voltage of the DGU-i is:

37 $$\:{I}_{ti}^{d}=-\sum\:_{j\in\:{\mathcal{N}}_{i}}\frac{{V}_{j}-{V}_{i}}{{R}_{ij}}+{I}_{Li}+\frac{{V}_{i}^{d}}{{R}_{Li}}-\frac{1}{{R}_{d1}}\left({V}_{i}-{V}_{i}^{d}\right)$$

38 $$\:{V}_{ti}={V}_{i}+{R}_{d2}\left({I}_{ti}-{I}_{ti}^{d}\right)$$

Equations (37) and (38) can achieve the control objective of the PBC approach. The control signal $\:{V}_{ti}$ can be synthesized based on the inductor current feedback (37). The outer control adjusts the inductor current based on the output voltage feedback (38).

However, due to the coupling term $\:\sum\:_{j\in\:{\mathcal{N}}_{i}}\frac{{V}_{j}-{V}_{i}}{{R}_{ij}}$, the entire parameters of the Microgrid are required to tune each controller. This requirement significantly limits the scalability and plug-and-play (PnP) capability of the DC Microgrid. To address these issues and enable decentralized control as well as PnP operation, starting from (1) since:

39 $$\:{I}_{ti}={C}_{ti}\frac{dVi}{dt}+{I}_{{L}_{i}}+\frac{1}{{R}_{Li}}{V}_{i}-\sum\:_{j\in\:{\mathcal{N}}_{i}}\frac{{V}_{j}-{V}_{i}}{{R}_{ij}}$$

Substituting (37) and (39) into (38), it yields:

40 $$\:{V}_{ti}={V}_{i}+{R}_{d2}\left({C}_{ti}\frac{dVi}{dt}+{I}_{{L}_{i}}+\frac{1}{{R}_{Li}}{V}_{i}-\sum\:_{j\in\:{\mathcal{N}}_{i}}\frac{{V}_{j}-{V}_{i}}{{R}_{ij}}-\left(-\sum\:_{j\in\:{\mathcal{N}}_{i}}\frac{{V}_{j}-{V}_{i}}{{R}_{ij}}+{I}_{Li}+\frac{{V}_{i}^{d}}{{R}_{Li}}-\frac{1}{{R}_{d1}}\left({V}_{i}-{V}_{i}^{d}\right)\right)\right)$$

41 $$\:{V}_{ti}={V}_{i}+{R}_{d2}\left({C}_{ti}\frac{dVi}{dt}+\frac{{V}_{i}}{{R}_{Li}}-\frac{{V}_{i}^{d}}{{R}_{Li}}+\frac{1}{{R}_{d1}}\left({V}_{i}-{V}_{i}^{d}\right)\right)$$

42 $$\:{V}_{ti}={V}_{i}+{R}_{d2}\left({C}_{ti}{\dot{V}}_{i}+\left(\frac{1}{{R}_{d1}}+\frac{1}{{R}_{Li}}\right)\left({V}_{i}-{V}_{i}^{d}\right)\right)$$

Define an error signal $\:{e}_{i}^{v}={V}_{i}^{d}-{V}_{i}$, and the result is the following controller:

43 $$\:{V}_{ti}={K}_{Di}{\dot{e}}_{i}^{v}+{K}_{Pi}{e}_{i}^{v}+{V}_{i}^{d}$$

where $\:{K}_{Di}$and $\:{K}_{Pi}$are the derivative and proportional gains given by:

$$\:{K}_{Di}={R}_{d2}{C}_{ti},\:{K}_{Pi}={R}_{d2}\left(\frac{1}{{R}_{d1}}+\frac{1}{{R}_{Li}}\right)$$

The PBC controller (43) is a PD type, and the output voltage $\:{V}_{i}\:$can be regulated by adjusting the damping virtual resistances $\:{R}_{d1}$ and$\:{\:R}_{d2}$. Motivated by^42,43^, we propose an integral error term to eliminate static errors. Thus, the PBC (43), after adding the integral term, can be written in the following form of the PID controller

44 $$\:{V}_{ti}={K}_{Di}{\dot{e}}_{i}^{v}+{K}_{Pi}{e}_{i}^{v}+{V}_{i}^{d}+{K}_{Ii}\int\:{e}_{i}^{v}dt$$

where $\:{K}_{Ii}$ is the integral gain. The control law in (44) enables decentralized voltage regulation at the PCC, relying solely on local voltage measurements and DGU-*i* parameters. This approach eliminates the need for system-wide parameters knowledge, simplifying the tuning process and ensuring it remains independent of the rest of the system. As a result, the design significantly enhances scalability and plug-and-play functionality, allowing the addition or removal of a DGU with only adjustments to its local controller without affecting the operation of neighboring units.

**Fig. 3:**
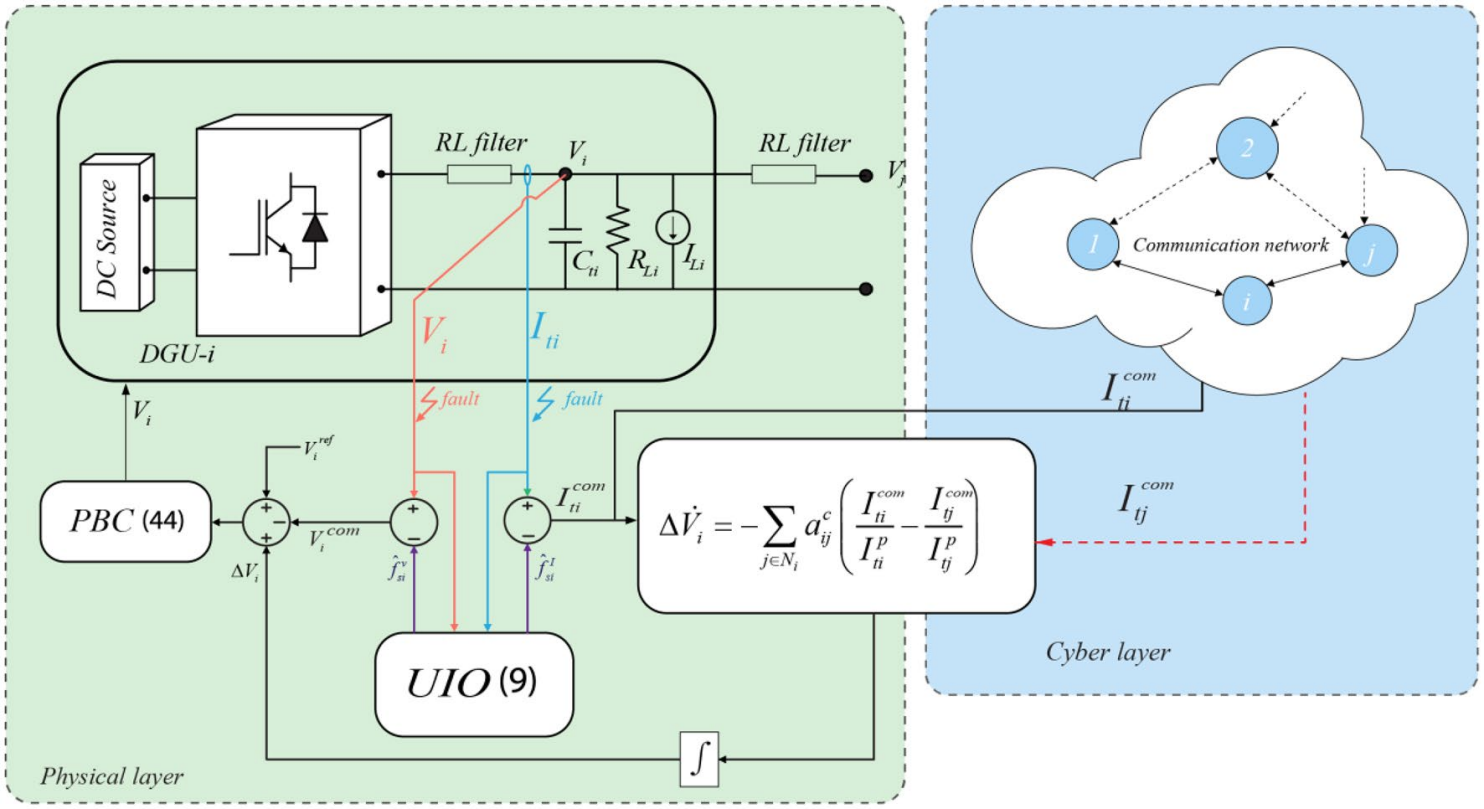
Schematic of the proposed fault-tolerant control framework for a DC Microgrid.

### Control reconfiguration

When a sensor fault occurs, the performance of the DC Microgrid will be seriously degraded, and even its stability can be jeopardized. For instance, a faulty current sensor at the level of one DGU will cause that fault to mislead the control system and, hence, the DGU to share less or more amount of power than intended, which could pose a stress or overload other neighboring DGUs. Therefore, to address the fault tolerance control of the voltage and current sensors, the fault effects can be tolerated using the fault estimation; the block diagram of the proposed sensor faults mitigation strategy for DC Microgrid is depicted in Fig. 3, The DGU-*i* employs a PID-based PBC controller to regulate the PCC voltage and consensus algorithm to achieve proportional current sharing when sensor faults are identified and estimated by the PI-UIO. The FTC scheme seamlessly takes effect to implement a control system reconfiguration by compensating directly in the control system the estimated fault (voltage or current). Where the estimated fault corrects the voltage or/and current measured by the faulty sensor. The fault compensation is expressed as:

45 $$\:{I}_{ti}^{com}={I}_{ti}-{f}_{si}^{I};{\:\:\:\:\:V}_{i}^{com}={V}_{i}-{f}_{si}^{V}$$

**Table 1:** DGUs electrical parameters.

/	Symbol	DGU-1	DGU-2	DGU-3	DGU-4	DGU-5	DGU-6
Local load	${R_{Li}}\;\left[ \Omega \right]$	10	4	15	6	6	10
	${I_{Li}}\;\left[ A \right]$	2	1.5	0.5	1	1.5	0
RLC	${R_{ti}}\;\left[ \Omega \right]$	0.2	0.3	0.5	0.1	0.11	0.21
	${L_{ti}}\;\left[ {mH} \right]$	0.18	0.2	0.3	0.22	0.23	0.17
	${C_{ti}}\;\left[ {mF} \right]$	0.22	0.19	0.25	0.17	0.18	0.22
Rated current	$I_{ti}^p\;\left[ A \right]$	9	15	6	12	5	4

where $\:{I}_{ti}^{com}$ and $\:{V}_{i}^{com}\:$are the reconfigured values provided to the control system. This way, the voltage/current value exists either locally in the voltage controller, and the consensus algorithms or information shared through the network will be the correct information rather than the measured value from the faulty sensor. Therefore, the normal operation of the DC Microgrid cannot be affected by sensor faults, which largely improves the resiliency and reliability of the whole system. Moreover, the sensor fault estimation is achieved without the use of communication and information exchange, thus enabling a rapid fault estimation and, consequently, fast fault mitigation.

## Simulation results

**Fig. 4:**
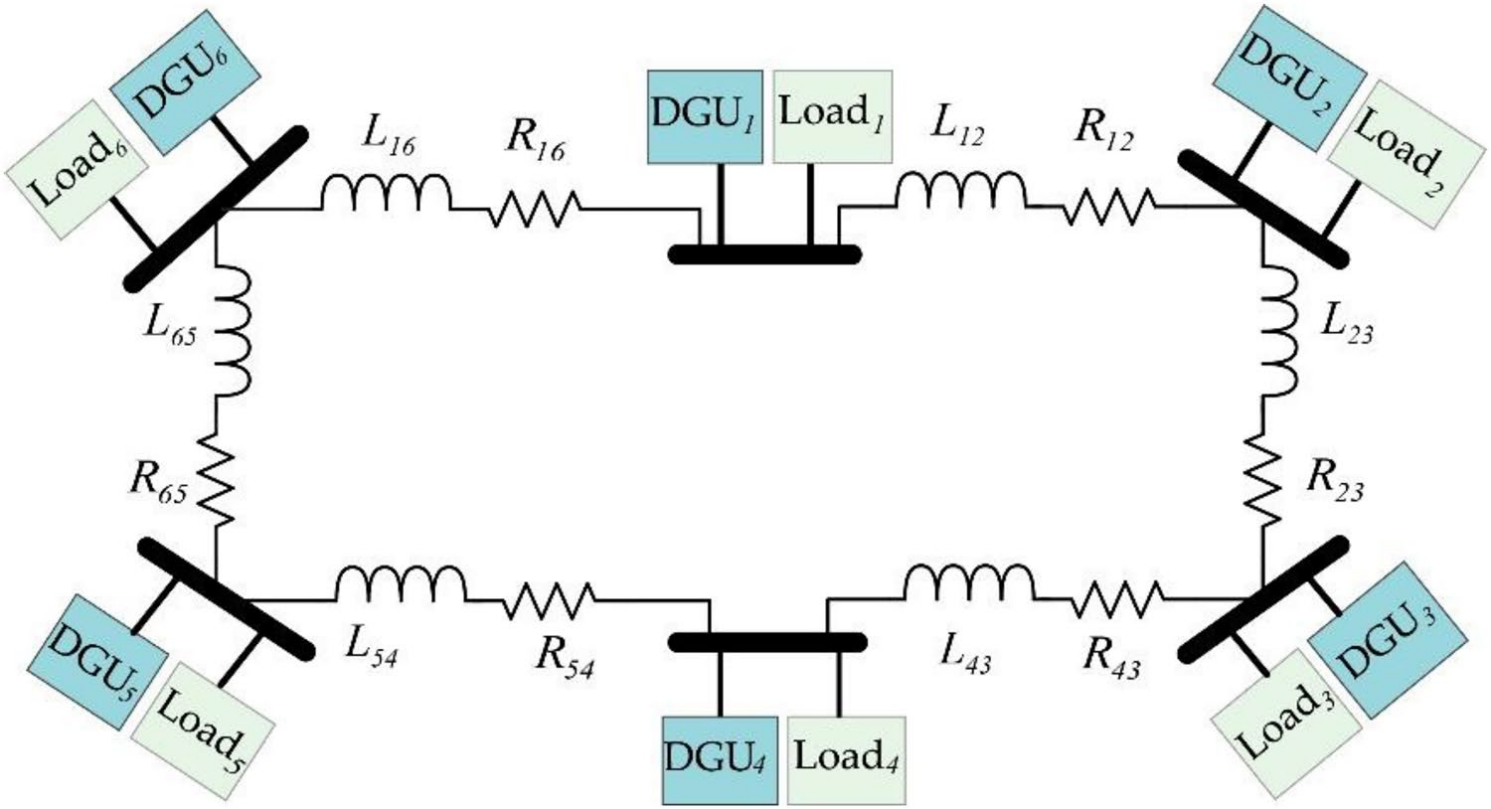
Topology of the test DC Microgrid consisting of six DGUs.

In this section, a simulation test model consisting of six (*N* = 6) DGUs as presented in Fig. 4, is built in Matlab/Simulink to validate the proposed sensor fault-tolerant control scheme. The system’s parameters are summarized in Tables 1 and 2.

**Fig. 5:**
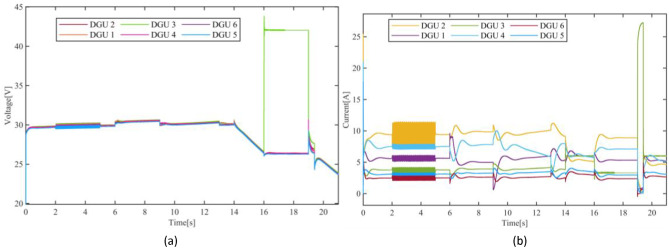
Impact of sensor faults on the dynamics of the adopted DC Microgrid: (**a**) Voltage dynamics, (**b**) Current dynamics.

**Fig. 6:**
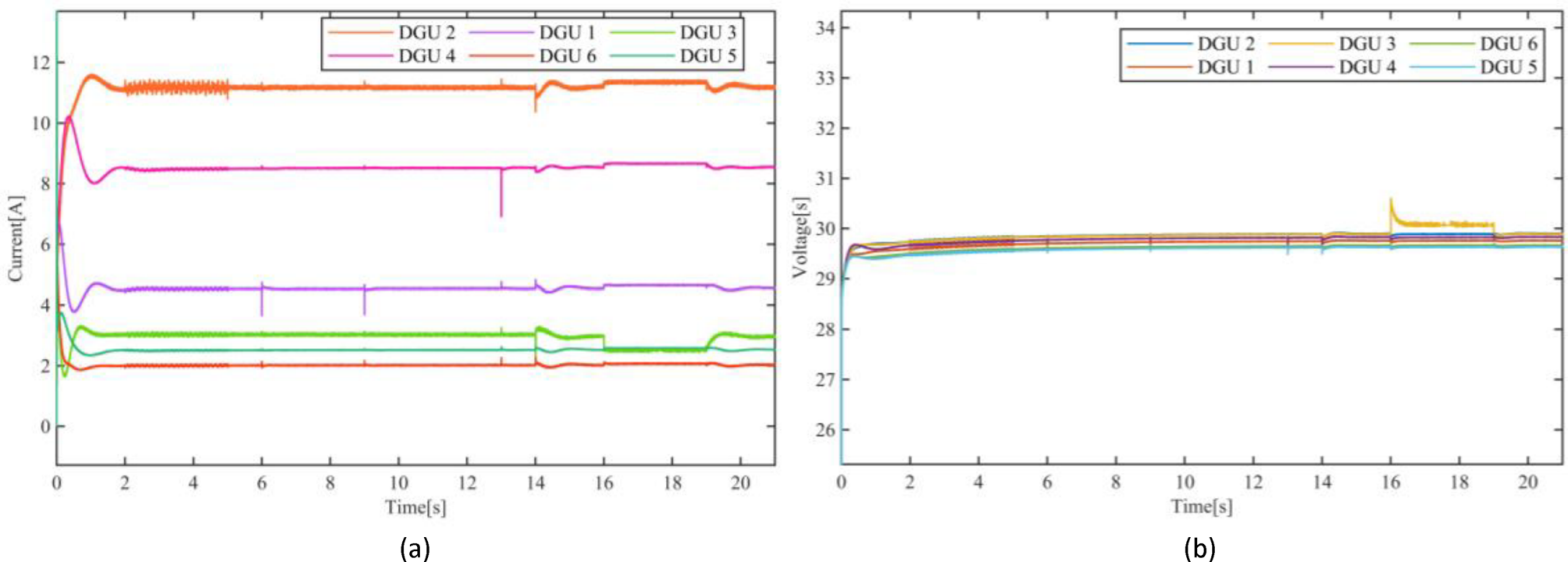
Results of the proposed FTC scheme: (**a**) Voltage dynamics, (**b**) Current dynamics.

**Fig. 7:**
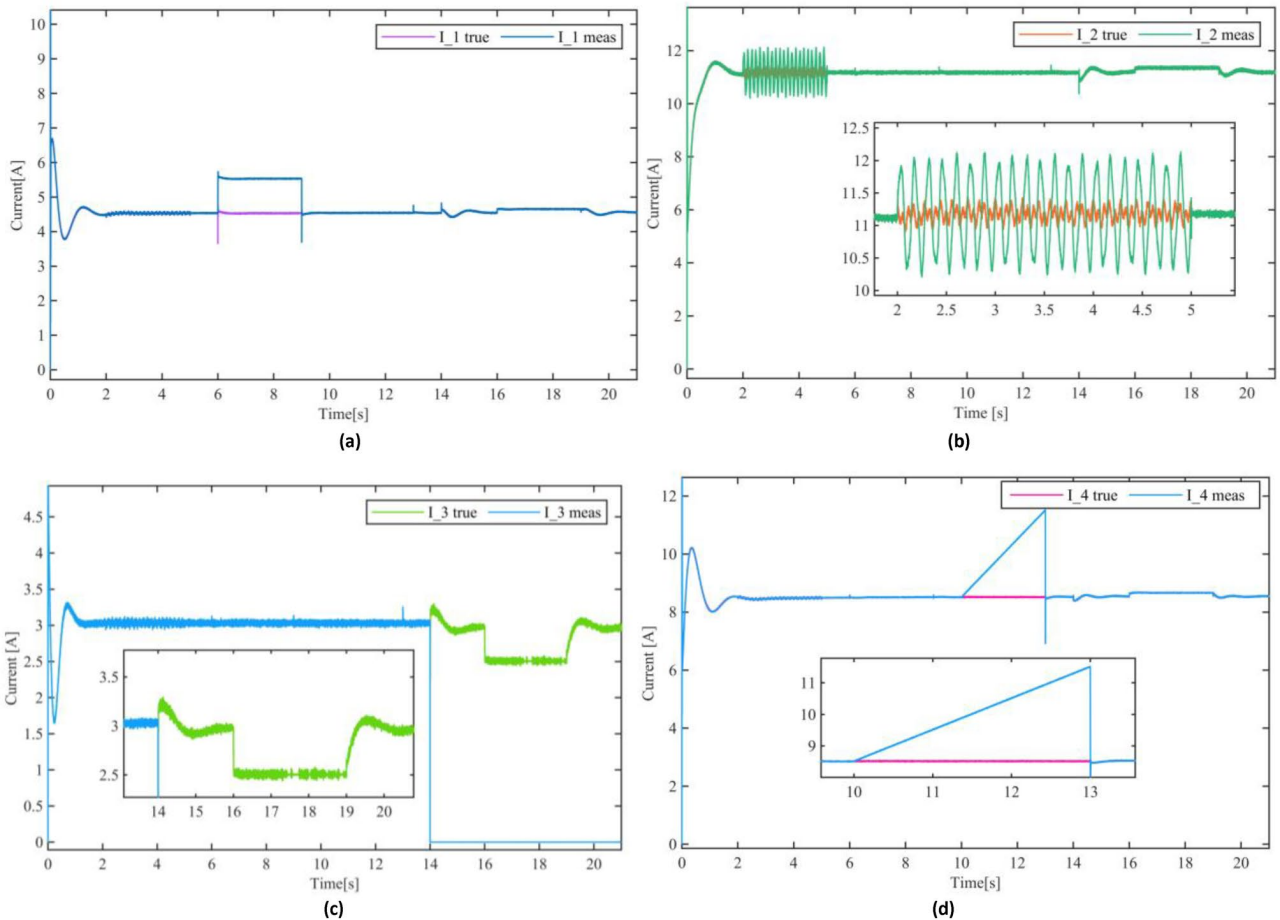
Measured and compensated currents dynamics of the DC Microgrid.

**Fig. 8:**
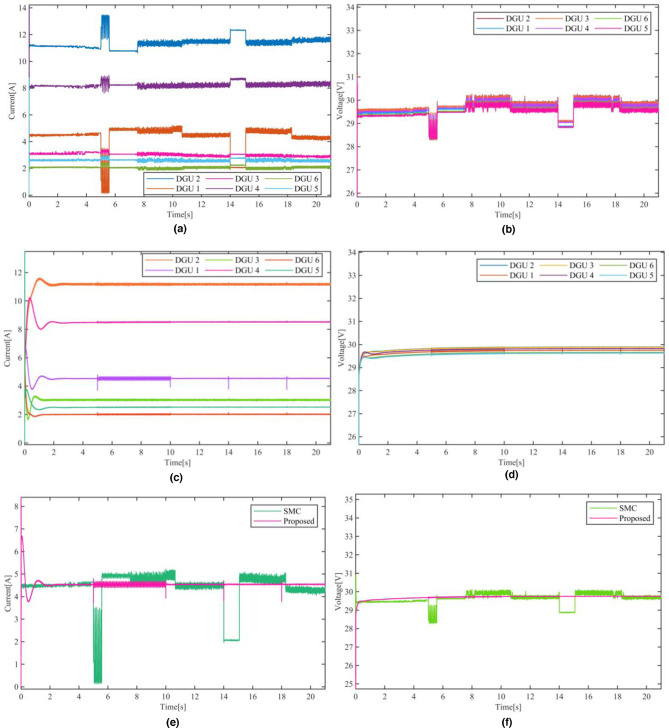
Currents and voltages responses of the adopted DC Microgrid controlled by two FTC schemes: (**a**) and (**b**) using the decentralized super-twisting based FTC^28^, (**c**) and (**d**) using the proposed FTC scheme. Subfigures (**e**) and (**f**) provide a comparative.

**Fig. 9:**
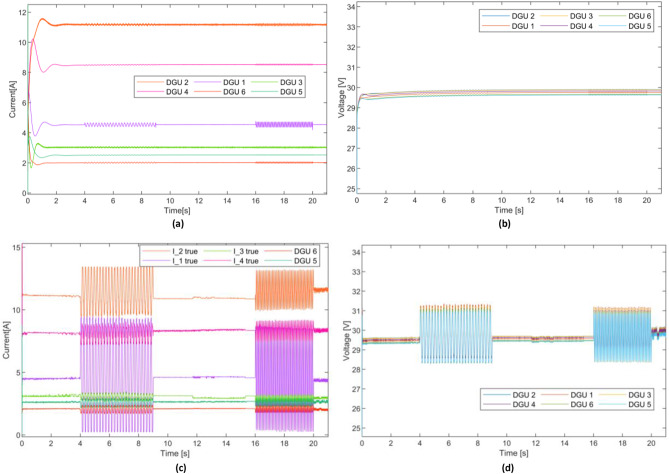
Currents and voltages responses of the adopted DC Microgrid controlled by two FTC schemes: (**a**) and (**b**) using the proposed FTC scheme, (**c**) and (**d**) using the decentralized super-twisting based FTC^25^.

A series of different sensor fault scenarios are set to occur at different instants to assess the effectiveness of the proposed scheme in mitigating different types of sensor faults and maintaining a reliable operational performance under diverse sensor fault conditions. First, a sensor fault is introduced to the voltage and current sensors of DGU-2 simultaneously during t={2,5}s defined as $\:{f}_{s2}^{I}=0.8\mathrm{sin}\left(44t\right)$ and $\:{f}_{s2}^{V}=1.5\mathrm{s}\mathrm{i}\mathrm{n}\left(113t\right)$. Subsequently, a constant sensor fault is applied simultaneously to voltage and current sensors of DGU-1 during t={6,9}s represented by $\:{f}_{s1}^{I}=1$ and $\:{f}_{s1}^{V}=-4$. During t={10,13}s, a simultaneous fault is set to occur in both current and voltage sensors of DGU-4, characterized by slop-shaped defined as $\:{f}_{s2}^{I}=t-10$ and $\:{f}_{s2}^{V}=-t+10$. Finally, a complete sensor failure is set to appear in DGU-3 at, affecting both voltage and current sensors simultaneously. Further, to validate the effectiveness of the proposed scheme fault-tolerant capability, the Plug-and-Play (PnP) operation of DGU was initiated under faulty sensors. Specifically, DGU-3 is removed and reconnected at t=16s and t=19s, respectively. Simulation results are presented in Figs. 5, 6 and 7, while comparative simulation results with existing methods are shown in Figs. 8 and 9.

Figure .5, depicts the current and voltage dynamics of the DGUs of the DC Microgrid without the application of fault compensation detailed in Section III.C. Fig. 5(b) illustrates the current of the five DGUs. In contrast, Fig. 5(a) depicts the voltages over time. As can be seen, the impact of the sensor fault severely compromised the DC Microgrid performance in both current sharing and voltage regulations. As evident, the impact of the sensor fault occurred in DGU-2 during t= {2,5}s is propagated to the other DGUs. More significantly, the volatile fluctuations and oscillations in the current completely degraded the current sharing, resulting in significant deviations, particularly during t= {6,16}s and during the reconnection of DGU3 at t=19s, where the current of DGU-3 significantly exceeded its capability and current sharing ratio. Similarly, the voltage dynamics highlight a failure to maintain proper regulation across the DGUs. These results underscore the detrimental impact of sensor faults on the DC Microgrids and vulnerabilities control systems sensors, particularly in DC Microgrid where accurate local measurements are indispensable. Hence underlines the necessity of a resilient

**Table 2:** DC Microgrid power lines parameters.

Line resistance	Symbol	$\:\left(\mathrm{1,2}\right)\:$	$\:\left(\mathrm{2,3}\right)$	$\:\left(\mathrm{4,3}\right)$	$\:\left(\mathrm{5,4}\right)$	$\:\left(\mathrm{6,5}\right)$	$\:\left(\mathrm{1,6}\right)$
	$\:{R}_{ij}\left[m\varOmega\:\right]$	68	49	76.8	62	55	60
Line inductance	$\:{L}_{ij}\left[\mu\:H\right]$	1.9	2.2	1.95	1.76	2.3	1.8

Figure6 illustrates the current and voltage dynamics of the DC Microgrid with the application of the proposed sensor fault tolerant control scheme, demonstrating remarkable effectiveness in mitigating the impact of the sensor faults. As evident in Fig. 6(a), the current sharing is successfully preserved despite the presence of various faults. Unlike the jeopardized performance seen in Fig. 5, the currents remain well-regulated, and the Microgrid sustains its operational stability throughout the duration of the faults. The proposed sensor fault tolerant control scheme not only ensures a resilient voltage regulation and current sharing but also exhibits rapid fault mitigation. For instance, at t=6s; the instant of the occurrence of faults in DGU-1, the PBC-based PI-UIO scheme immediately tolerated the faults in both current and voltage sensors, inducing a transient spike of approximately 15% in the current magnitude settled within approximately 5ms, restoring system stability. Fig. 7(a) compares the faulty measured current and the reconfigured $\:{\mathrm{I}}_{\mathrm{t}1}^{\mathrm{c}\mathrm{o}\mathrm{m}}$for DGU-1. Similar observations can be made at t=10s where the simultaneous sensor faults in DGU-4 initially introduce a transient disturbance. The FTC scheme efficiently compensates for these faults, achieving quick stabilization with negligible impact on current-sharing accuracy and voltage regulation; Fig. 7(d) displays the comparison between the faulty measured current and the reconfigured $\:{I}_{t4}^{com}$ for DGU-4. Notably the proposed scheme exhibits an exceptional performance handling the severe faults scenarios including the complete simultaneous sensor failure at t=14s in DGU-3 depicted in Fig. 7(c), responding instantaneously, mitigating the faults without disrupting the Microgrid overall performance. Such outstanding results highlight the scheme resiliency in addressing most critical sensor faults. The faulty measured current versus the without disrupting the Microgrid overall performance. Such outstanding results highlight the scheme resiliency in addressing most critical sensor faults. The faulty measured current versus the reconfigured $\:{\mathrm{I}}_{\mathrm{t}3}^{\mathrm{c}\mathrm{o}\mathrm{m}}$ is illustrated in Fig. 7(c). The significance of the proposed scheme is further evident during the PnP operation of DGU-3 between t=16s and t=19s.

reconfigured $\:{\mathrm{I}}_{\mathrm{t}3}^{\mathrm{c}\mathrm{o}\mathrm{m}}$ is illustrated in Fig. 7(c). The significance of the proposed scheme is further evident during the PnP operation of DGU-3 between t=16s and t=19s.

Despite the persistent complexity of disconnection and reintegrating, the proposed scheme achieves a seamless PnP operation, maintaining accurate current sharing and voltage regulation with a brief transient in the voltage of approximately 16% without observable disruptions in the overall Microgrid seen in Fig. 6(b). These results unequivocally demonstrate the exceptional performance of the proposed sensor FTC scheme, underscoring its ability to adapt to the dynamic DC Microgrid reconfiguration while tolerating sensor faults in real-time. This adaptability ensures key operational goals including consistent current sharing, rapid transient response, and precise voltage regulation. These critical requirements are essential for maintaining the reliable and stable operation of DC Microgrids under varying conditions and potential sensor failures. More importantly, the achieved resiliency is realized without relying on communication, instead utilizing only local measurement thus reinforcing the practicality and scalability of the proposed scheme DC Microgrid.

To illustrate the superiority of the proposed sensor fault tolerant control scheme, a comparative analysis is conducted in this part with the super-twisting sliding mode controller sensor FTC scheme reported in^28^. It is important to note that, as discussed in the literature review, the existent sensor FTC schemes rely on communication for fault mitigation, making a direct comparison with the proposed sensor FTC communication-free strategy, less appropriate. Therefore, the decentralised method in^28^ is selected for a fair comparison.

Figure.8 illustrates a comparative evaluation between the decentralized sensor FTC suggested in^28^—shown in Figs. 8(a) and 8(c)—and the proposed scheme—shown in Figs. 8(b) and 8(d). Since the super-twisting-based FTC scheme in^28^ does not handle the occurrence of simultaneous faults, only a time-varying with a bias and a bias faults set to occur in the current sensor during t={5,10}s and t={14,18}s defined as $\:{f}_{s1}^{I}=1\mathrm{sin}\left(62t\right)-1.5$ and $\:{f}_{s1}^{V}=-1$, respectively. As evident in Fig.8, the proposed sensor FTC strategy significantly outperforms the approach in^28^ in terms of fault detection speed, fault mitigation, and overall Microgrid stability. When the fault is injected at t=5s, the decentralized scheme exhibits a detection and isolation delay of 0.57s, during which the fault propagates and disrupts both the current sharing and the voltage regulation; this is showcased as biased oscillations in the voltage of the affected DGU as well as neighbouring DGUs thus jeopardizes overall stability. Even after the mitigation of the fault the current sharing accuracy remains degraded as seen in Fig. 8(e). In contrast, the proposed method swiftly isolates and tolerates the faults, preventing their propagation and safeguarding control system stability. The transient response is negligible, with swift stabilization attained within 5ms seconds, ensuring that current distribution and voltage regulation are preserved; comparative analysis is presented in Table III. Furthermore, the results underscore the superiority, resilience, and flexibility of the proposed scheme, offering faster and more reliable communication-free fault-tolerant control and greater scalability.

Both the proposed scheme and the SMC-based scheme proposed in^28^ operate at the same execution rate of 5 μs, which is also the simulation sampling time T_s_. However, the key difference lies in how each scheme responds to sensor faults within this rate. In the proposed method, fault accommodation and mitigation occur instantaneously, meaning the controller reacts immediately upon fault occurrence. As a result, the fault recovery time presented in the comparison table III primarily represents the settling time after the initial overshoot, rather than a delay in fault handling. On the other hand, the SMC-based scheme proposed in^28^ requires approximately 0.57 s to detect and compensate for the fault, leading to a prolonged deviation in the system’s current and voltage responses. This distinction is clearly observable in Figs. 8(e) and 8(f), where the waveforms under the proposed scheme exhibit minimal disturbance, while the SMC-based scheme shows significant transients during the fault period.

To further validate the superiority of the proposed scheme, an additional test scenario is presented in Fig. 9. In this test a time-varying sinusoidal sensor faults defined as $\:{\mathrm{f}}_{\mathrm{s}1}^{\mathrm{I}}=2\mathrm{sin}\left(31\mathrm{t}\right)\:\:$and $\:{\mathrm{f}}_{\mathrm{s}1}^{\mathrm{I}}=1.5\mathrm{sin}\left(62\mathrm{t}\right)\:$—commonly employed in prior studies^31,32^— are set to occur in DGU-1 current sensor during t={4,9}s and t={16,20}s. The test is particularly important due to the periodic nature of the sinusoidal faults as it introduces a challenge for a residual-based fault detection schemes such as the one in^28^. These schemes rely on comparing a residual signal to a predefined threshold. However, when the fault is sinusoidal, the residual signal also becomes periodic—crossing above and below the threshold— rendering it potentially confusing, and in the case of^28^, the slow detection time results in a complete failure to detect and identify the fault. This limitation is clearly depicted in Figs. 9(c) and 9(d). The decentralised FTC scheme fails to detected and identified the injected sensor faults, consequently is unable to tolerate them, as a result the faults propagated through the Microgrid, negatively affecting the operation of the other DGUs. This leads to degraded current sharing and deviation in voltage across the Microgrid. In contrast, the proposed sensor FTC successfully detect, identify and compensates for sinusoidal sensor faults in real time, owing to its capability to estimate faults directly as illustrated in Fig. 9(a) and 9(b). The proposed fault tolerant scheme allows the controller to maintain stable operation and proper performance, even under sudden challenging and time-varying faults conditions.

## Real time simulation results

**Fig. 10:**
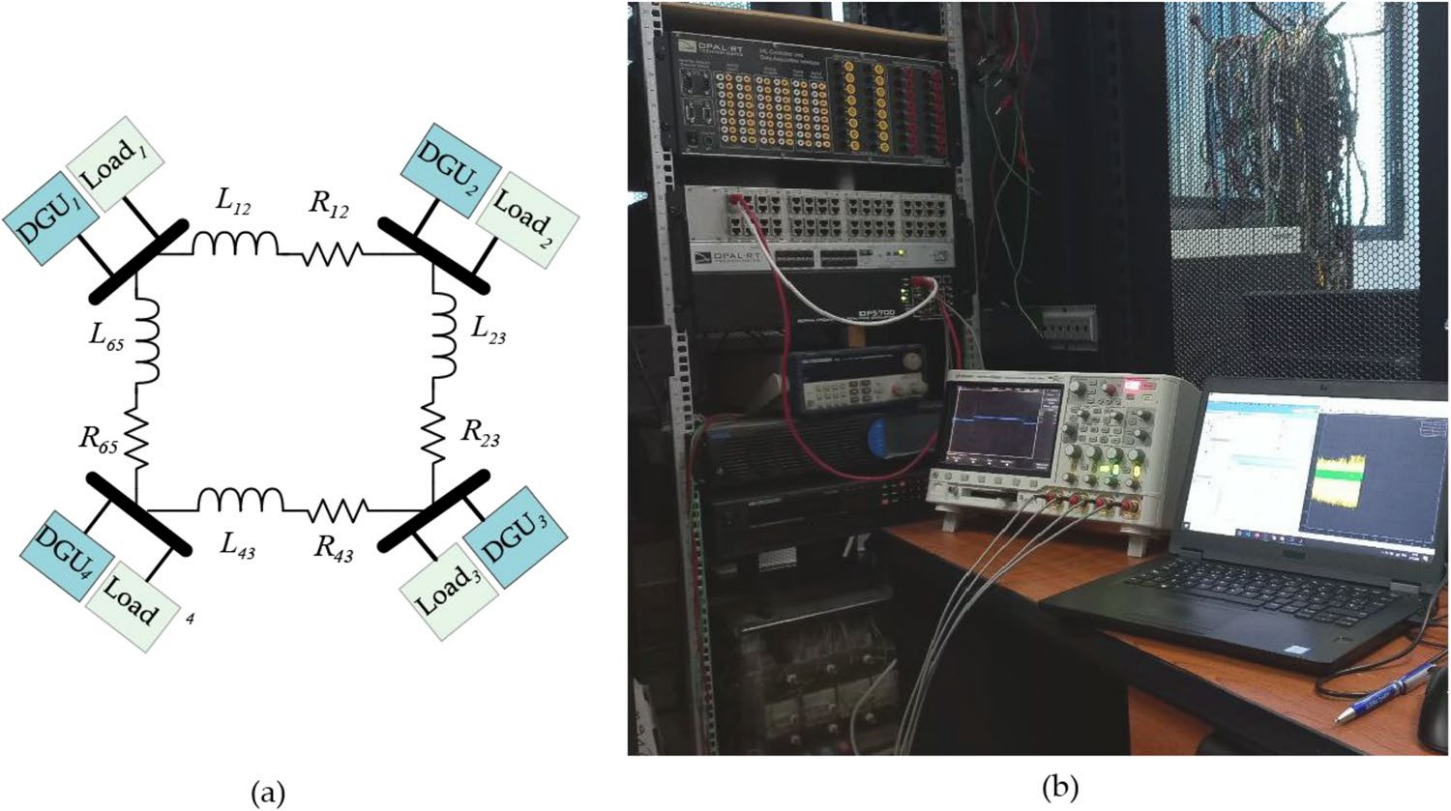
Real-time simulation framework used for validation: (**a**) DC Microgrid electrical structure of the test model setup, (**b**) OPAL-RT-based real-time simulation setup used to evaluate the proposed fault-tolerant control scheme.

To further evaluate the feasibility and resilience of the proposed sensor fault-tolerant control scheme, real-time tests were conducted using the OPAL-RT OP5700 system, as illustrated in Fig. 10(b). The experimental setup comprises a host PC used to visualize and monitor the real-time simulation, which communicates with the OP5700 real-time target via a TCP/IP interface. The real-time validation was performed on a DC Microgrid test system consisting of four distributed generation units (DGUs) interconnected in a ring topology, as shown in Fig. 10(a). The entire model was implemented on the OPAL-RT OP5700 hardware-in-the-loop (HIL) platform, where all control algorithms were executed on the same real-time computational target using the RT-LAB environment to ensure identical execution conditions across all controllers.

The simulation was carried out using a fixed-step solver with a sampling period of $\:{T}_{s}=5\mu\:s$, and each DGU was equipped with the proposed fault-tolerant control scheme. The local load resistances were set to $\:{R}_{L1}=4\left[\varOmega\:\right]{,R}_{L2}=4\left[\varOmega\:\right]{,R}_{L3}=4\left[\varOmega\:\right]{,\mathrm{a}\mathrm{n}\mathrm{d}\:R}_{L4}=4\left[\varOmega\:\right]$. while the power line parameters were selected as reported in Table 2.

**Fig. 11:**
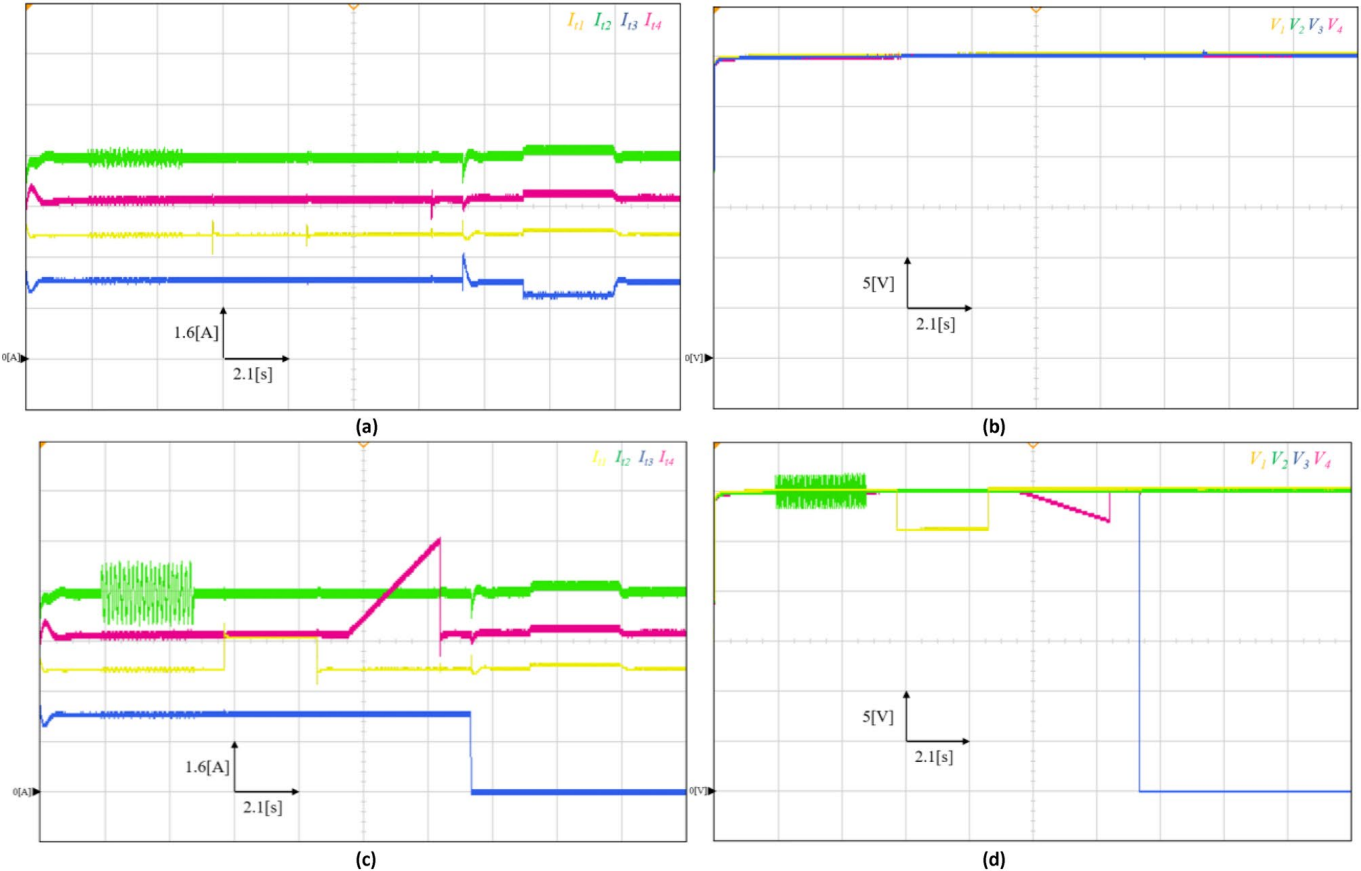
Real-time results: (**a**) Raw currents measurement, (**b**) Raw voltages measurement, (**c**) Faulty measured currents, (**d**) Faulty measured voltages.

For consistency with the simulation study, the same sensor fault scenarios are introduced in the experimental setup.

Figure 11 depicts the raw and measured voltage and current dynamics of the DC Microgrid under various fault scenarios. As can be seen, the real-time results confirm the resiliency of the proposed sensor fault tolerant control scheme in effectively mitigating the impact of various sensor faults occurring across all DGUs while preserving the system stability and accurate current sharing among DGUs. As observed in Figs. 11(c) and 11(d), the sensor faults corrupt the measured currents and voltages of the DGUs. However, in contrast, the raw currents/voltages remain unaffected, demonstrating the proposed FTC scheme’s capability to correctly tolerate sensor faults. Notably, the PnP capabilities of DGUs are also investigated under the presence of complete sensor failure. As shown in Figs. 11(c) and 11(d), both voltage and current sensors of DGU-3 experience a complete failure at t = 14s. Shortly after that, at t=16s, DGU-3 is plugged out from the Microgrid, altering the electrical structure. As a result, the current sharing is re-established among DGU-1, 2, and 4, and DGU-3 supplies only its local load. At t=19s, when DGU-3 is reconnected, the FTC scheme successfully restores current sharing among all DGUs without destabilizing the system.

These real time simulation results demonstrate the proposed scheme’s ability to maintain voltage regulation and current sharing despite the presence of sensor faults. By autonomously mitigating potential sensor faults and failures, the FTC scheme ensures that the DC Microgrid continues to meet its operational objectives without requiring additional communication links or redundant hardware. This underscores its practical applicability in real-world DC Microgrid implementations, where sensor faults failures are common challenges.

## Conclusion

This paper proposes a real-time fault-tolerant control framework for DC Microgrids under sensor faults, ensuring accurate voltage regulation and current sharing. To this end, a local proportional-integral unknown input observer dynamically reconfigures both the primary voltage passivity-based control and the secondary distributed consensus-based layer. This dual adjustment allows the system to systematically mitigate sensor faults and failures while ensuring an autonomous and communication-free fault-tolerant control. Notably, the proposed scheme achieves instantaneous fault detection and reconfiguration in real-time, with a fault accommodation time of just 5 µs and a transient duration of only 5 ms due to controller reconfiguration, ensuring uninterrupted system performance. Furthermore, the scheme is designed to facilitate plug-and-play operation and enhance the scalability, reliability, and resilience of the DC Microgrid, even under faulty sensor conditions. For a fair comparison, the framework was evaluated against a recent decentralized, communication-free, resilient control method. The results demonstrate that the proposed scheme outperforms existing methods in performance and fault mitigation time. Extensive Simulation and real-time simulation results further validate the effectiveness and superiority of the proposed fault-tolerant control approach. Future research directions include investigating cyber-attacks on the communication layer, with the goal of enhancing the overall security and resilience of the distributed control architecture. Another important direction involves experimental validation on power-hardware platforms, bridging the gap between real-time simulation and field deployment. Finally, the extension of the proposed methodology to hybrid AC/DC Microgrids and large-scale interconnected systems represents a promising research avenue, enabling scalable and resilient fault-tolerant control architectures for future smart grids.

## Data Availability

The datasets used and/or analyzed during the current study are available from co-author Prof. Abdelaziz Rabehi (Abdelaziz.rabehi@univ-djelfa.dz) on reasonable request.
